# Simultaneous exposure to vinylcyclohexene and methylmercury in *Drosophila melanogaster*: biochemical and molecular analyses

**DOI:** 10.1186/s40360-019-0356-0

**Published:** 2019-12-19

**Authors:** Bruna Candia Piccoli, Ana Lúcia Anversa Segatto, Cláudia Sirlene Oliveira, Fernanda D’Avila da Silva, Michael Aschner, João Batista Teixeira da Rocha

**Affiliations:** 1 Departamento de Bioquímica e Biologia Molecular, Centro de Ciências Naturais e Exatas, Universidade Federal de Santa Maria, Santa Maria, RS Brazil; 2 Programa Pós-Graduação Stricto Sensu em Biotecnologia Aplicada a Saúde da Criança e do Adolescente, Instituto de Pesquisa Pelé Pequeno Príncipe, Curitiba, Paraná Brazil; 3 Faculdades Pequeno Príncipe, Curitiba, Paraná Brazil; 4 Department of Molecular Pharmacology, Albert Einstein College of Medicine, Bronx, NY USA

**Keywords:** Oxidative stress, Inflammatory response, Xenobiotic, Alternative model, *Drosophila melanogaster*

## Abstract

**Background:**

Exposure to vinylcyclohexene (VCH) and methylmercury (MeHg^+^) can induce oxidative stress and gene modulation. Several studies have been evaluating the effects of VCH and MeHg^+^, but little is known about interactive effects between them. This work aimed to assess the exposure and co-exposure effects of MeHg^+^ and VCH on oxidative stress and gene modulation in *Drosophila melanogaster*.

**Methods:**

Reactive species production, glutathione S-transferase (GST) and acetylcholinesterase (AChE) activities were evaluated after exposure and co-exposure to VCH (1 mM) and MeHg^+^ (0.2 mM) for one or three days in the head and body (thorax and abdomen) of flies. The expression of genes related to redox state and inflammatory response was evaluated after exposure and co-exposure to VCH and MeHg^+^ for three days.

**Results:**

Survival decreased only in flies co-exposed to VCH and MeHg^+^ for three days. All treatments increased total reactive species production after one day of exposure. However, no significant changes were observed in the head after three days of exposure. One day of exposure to VCH caused an increase in the head GST activity, whereas MeHg^+^ induced an increase after three days of exposure. Regarding the body, all treatments increased GST activity after one day of exposure, but only the flies exposed to MeHg^+^ presented an increase in GST activity after three days of exposure. Treatments did not alter AChE activity in the head. As for gene expression, there was a significant increase in the Relish transcription factor gene in the flies’ body, but Nrf2, Keap1, Jafrac1, TrxR1, and NF-κβ were not altered.

**Conclusion:**

The results suggest that exposure to VCH and MeHg^+^ induce oxidative stress and activation of an inflammatory response in fruit flies.

## Background

The toxicity of environmentally relevant contaminants has been commonly determined individually. However, in “real life scenarios” the population is exposed simultaneously to more than one xenobiotic [1, 2]. Toxicants are widely distributed in the environment, and those found at high levels are mostly associated with anthropogenic activities [1, 3]. The plastic derivative vinylcyclohexene (VCH) and the ubiquitous metal, mercury (Hg) are examples of environmental contaminants.

VCH is used commercially as an epoxy resin diluent used in the production of plastic, rubber, and pesticides [4]. Of particular environmental and toxicological concerns, VCH is found at high concentrations in wastewater derived from the production of hydroxylated liquid polybutadiene [5]. VCH exposure may occur through inhalation, ingestion, or dermal contact [6, 7]. Once in the body, VCH can be oxidized by cytochromes P450, and its double bonds are transformed into epoxy groups [8]. The liver has two cytochromes P450 isoforms, 2A and 2B, which bioactivate VCH into its mono (4-vinylcyclohexene 1,2 epoxide or 4-vinylcyclohexene 7,8 epoxide) and diepoxide (VCD - 4-vinylcyclohexene diepoxide) metabolites, respectively [9]. Also, the ovaries have a cytochrome P450 isoform, 2E1, which oxidizes the two double bonds, forming the diepoxide metabolite [10]. Due to the electrophilicity of the epoxide groups, they have an affinity for nucleophilic centers, such as thiol groups [11, 12]. Therefore, thiol-containing proteins can be targeted by epoxides in cells representing one of the mechanisms of VCH metabolites toxicity. In rodents, VCH and its metabolites have been associated mainly with ovarian damage characterized by the death of primary and primordial follicles [13–15]. Besides, exposure to VCH or its epoxides has been shown to cause toxic effects in various organs in rodents [15–19].

Another xenobiotic is mercury (Hg), a highly toxic metal and important environmental contaminant that has no function in living organisms [20–22]. Contamination by Hg has increased due to anthropogenic activities [23]. Among the chemical forms of mercury, the major health concerns are related to the organic forms, i.e., methylmercury (MeHg^+^). MeHg^+^ is found in the aquatic environment as a product of Hg^2+^ methylation by microorganisms [24], once formed MeHg^+^ bioaccumulates in the food chain [25–28]. Consequently, the consumption of piscivorous fish by humans is of concern since they are a relevant source of MeHg^+^ [20–22, 29, 30]. Since MeHg^+^ is a soft electrophile, it has a high affinity for soft nucleophiles, i.e., thiol [31–33] and selenol groups, which can be found in proteins [34, 35]. Moreover, thiol groups are found in low molecular mass molecules, such as reduced glutathione (GSH) and cysteine. The affinity of MeHg^+^ for thiol groups is extremely high, and practically no free MeHg^+^ is found in living cells. Indeed, MeHg^+^ in the biological medium is bound to cysteine, GSH, or target proteins. In mammals, MeHg^+^ is transported bound to cysteine or GSH [36, 37]. Glutathione S-transferases (GSTs) are a family of detoxifying enzymes. GSTs catalyze the conjugation of GSH with electrophilic compounds to be neutralized, transported, and eliminated. Several studies have established the involvement of GST in the conjugation of GSH with MeHg^+^ [33, 38] and VCD [39–41].

The use of *Drosophila melanogaster* in toxicological studies has increased [42–52] given the genome of flies has homology to the human genome [53], thus making it a highly predictive model of toxicity in vertebrates. *D. melanogaster* has numerous thiol-containing proteins involved in redox signaling [54] and five selenoproteins [55–59]. One of these selenoproteins is selenophosphate synthetase [55], which catalyzes the synthesis of monoselenophosphate. The other four identified selenoproteins are glycine-rich selenoprotein (SelG) [56], selenoprotein birthday (Bthd) [57], ring canal kelch protein (Kel) [58], and glucose dehydrogenase (Gld) [59]. Thus, the toxicity of VCH and MeHg^+^ in *D. melanogaster* may be secondary to the inactivation of thiol- and/or selenol-containing proteins.

The majority of the toxicological studies investigate the isolate toxicity of a given contaminant [14, 60–63]. Studies comparing the effect of exposure to two or more toxicants are timely and meritorious as to characterize potential interactions between environmental contaminants. Here, we hypothesize that co-exposure to MeHg^+^ and VCH may have an overlapping mechanism of toxicity via thiol oxidation. Consequently, MeHg^+^ and VCH coexposure may cause synergistic or additive effects. Accordingly, we used *D. melanogaster* as a model to study the potential toxicological interactions between VCH and MeHg^+^. Markers of electrophilic toxicity, such as reactive species production, GST, and acetylcholinesterase (AChE) activity were determined after exposure and co-exposure to VCH and MeHg^+^ for one or three days. Furthermore, we have also assessed the expression of genes related to oxidative stress and found that some of them were modulated after VCH and MeHg^+^co-exposure.

## Methods

### Chemicals

All chemicals were of analytical grade. 4-vinylcyclohexene (99%), methylmercury chloride, 1-chloro-2,4-dinitrobenzene (CDNB), 5,5′-dithiobis (2-nitro-benzoic acid) (DTNB), acetylthiocholine iodide, and 2,7-dichlorofluorescein diacetate (DCFDA) were purchased from Sigma Aldrich (St. Louis, MO, USA).

### Stock and culture

Flies (*D. melanogaster*) were produced by the Toxicological Biochemistry Laboratory of the Universidade Federal de Santa Maria (UFSM), Brazil. Stocks were maintained and reared into a medium composed of cornmeal (1%), sucrose (1%), powdered milk (1%), agar (1%), yeast extract (2%), and nipagin (0.08%), mixed and cooked with distilled water (200 mL). Temperature and relative humidity were constant at 23 °C and 60%, respectively, under 12 h dark/light cycle conditions.

### Concentration curves of MeHg^+^ and VCH toxicity

**Fig. 1:**
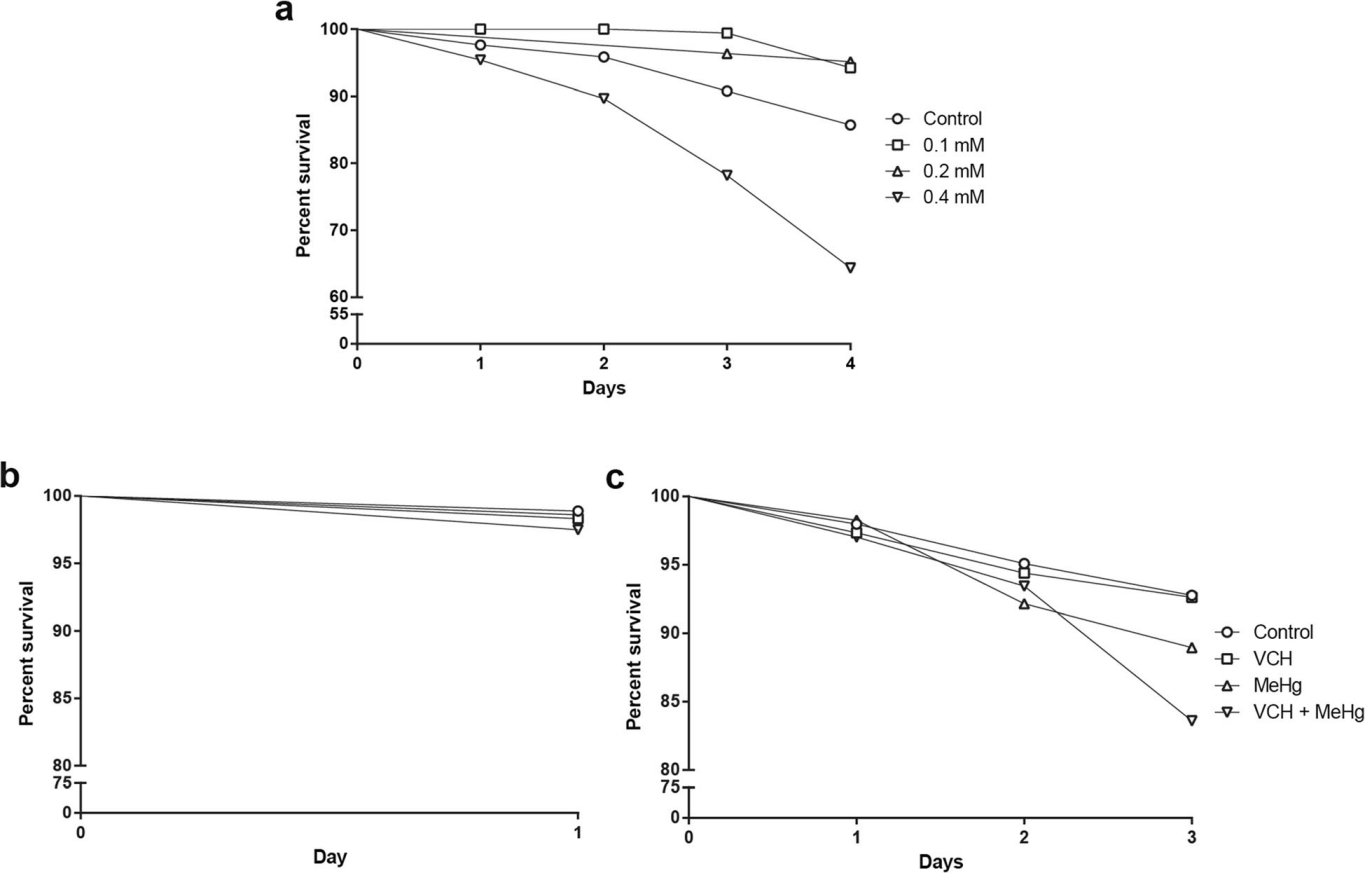
Kaplan-Meier percent survival of flies after exposure to VCH and MeHg^+^. Percent survival of flies after exposure to different concentrations of MeHg^+^ (0 - 0.4 mM) for four days (**a**). Percent survival of flies after exposure and co-exposure to VCH and MeHg^+^ for one (**b**) or three-days (**c**). Results were analyzed by Logrank test for trend and were considered significantly different when *p* < 0.05.

To determine the concentration to be used in the co-exposure treatment, 30 flies (both gender), three-days-old, were exposed to different concentrations of MeHg^+^ (0, 0.1, 0.2, and 0.4 mM) diluted in ethanol (0.1%). The experimental procedure consisted of three independent replicates for each concentration tested. The number of dead flies was registered daily for four days (Fig. 1a). The highest concentration of MeHg^+^ that did not alter flies' survival (0.2 mM) was selected to study the coexposure with VCH. The VCH concentration was selected based on the study performed by Abolaji et al. [44], where 1 mM was the highest concentration of VCH that did not alter the flies' survival after five days of exposure.

### Co-exposure to VCH and MeHg^+^

The control group was raised in medium containing ethanol (0.1%), VCH group in 1 mmol VCH/L of fly food, MeHg^+^ group in 0.2 mmol MeHg^+^/L of fly food and VCH + MeHg^+^ group in 1 mmol VCH/L + 0.2 mmol MeHg^+^/L of fly food. Because of the strong affinity of MeHg^+^ for thiol groups [34, 37] it is expected that the MeHg^+^ will react to the thiol-containing proteins of the diet, mainly the yeast proteins (data are not shown), forming an R-S-HgMe complex (R = protein), which mimics the intake of this compound by vertebrates [64–68]. In fact, the MeHg^+^ in fish muscle is found almost exclusively bound to cysteinyl residues of proteins [66]. We also speculated that MeHg^+^ did not interact with the VCH in the medium because it was associated with food protein. Thirty three-days-old flies were placed into the vials containing the experimental medium and treated for one or three days. At the end of exposure periods (one or three days), flies had four and six-days-old, respectively. The number of dead flies was registered every day. Each experimental procedure consisted of twelve independent replicates.

### Negative geotaxis

The negative geotaxis assay was carried out to determine the locomotor performance of flies after exposure and co-exposure to VCH and MeHg^+^ for one or three days. For this assay, ten flies from each group were cryoanesthetized and placed in vertical glass columns with 15 cm of length and 1.5 cm of diameter with a marking at the 6th cm. After 20 min (recovery from ice exposure), the flies were tapped to the bottom of the column by a gentle beat, and after 6 s, the number of flies that climbed up the mark was recorded. This assay was independently performed twelve times for each treatment. The number of flies at the top in each replicate was expressed as a percentage of the total number of flies.

### Biochemical analyses

#### Preparation of samples

At the end of the treatments, twenty flies were cryoanesthetized, and their heads were separated from the thorax and abdomen (hereafter named as the body). Heads and bodies were homogenized with 200 μL of 100 mM potassium phosphate buffer pH 7.4 and centrifuged at 13,000 rpm for 10 min at 4 °C. Subsequently, the supernatant was separated from the pellet, and the concentration of protein was determined in the spectrometer Spectra Max at 280 nm. The protein concentration was adjusted to 0.4 mg/mL for head and 0.3 mg/mL for body samples. The samples were used for the determination of AChE and GST activities, and reactive species (both oxygen and nitrogen) production. AChE levels in the body were below the level of detection.

#### Assessment of dichlorofluorescein diacetate oxidation

The DCFDA assay, which is a general index of oxidative stress, was determined as described by Pérez-Severiano et al. [69]. Briefly, potassium phosphate buffer pH 7.4 (75 mM) and DCFDA (5 μM) were mixed with 5 μL of sample, and the fluorescence was monitored using a spectrophotometer Spectra Max plate reader, at 488/525 nm of excitation and emission for 25 min with an interval of 30 s. Results were expressed as the mean of fluorescence intensity unit per minute.

#### Determination of glutatione S-transferase activity

GST activity was determined according to Habig et al. [70]. The system consisted of potassium phosphate buffer pH 7.4 (70 mM), ethylenediaminetetraacetic acid (1 mM), GSH (3.20 mM), and 100 μL of sample; then the reaction was started by adding CDNB (0.80 mM). The reaction was monitored using a spectrophotometer Spectra Max plate reader, at 340 nm for 25 min with an interval of 30 s. Results were expressed as the mean of absorbance of GSH-CDNB conjugate per minute.

#### Determination of acetylcholinesterase activity

AChE activity was determined according to Ellman et al. [71]. The system consisted of potassium phosphate buffer pH 7.4 (10 mM), DTNB (1 mM), acetylthiocholine (0.8 mM), and 30 μL of sample. The activity was monitored at 412 nm for 10 min with an interval of 2 min. Results were expressed as the mean of acetylthiocholine hydrolyzed in nmol per mg protein per minute.

### RNA extraction and reverse transcription-quantitative polymerase chain reaction (RT-qPCR)

Total RNA was extracted from ten heads or bodies of flies using Trizol® according to the manufacturer’s protocol. RNA present in the samples was quantified in Nanodrop2000™ and visualized in 1.5% agarose gel. RNA (1 μg) was treated with DNase I (Invitrogen) according to the manufacturer’s specifications. cDNA was synthesized using iScript™ cDNA Synthesis kit according to the manufacturer’s protocol. The amount of RNA was quantified in Nanodrop2000™, and 2.5 ng/μL was used in RT-qPCR. The primer sequences used in this study were Nuclear factor-erythroid 2-related factor 2 (Nrf2), Kelch-like erythroid cell-derived associated protein 1 (Keap1), Nuclear factor-κB (NF-κB) activating protein-like, Jafrac1, thioredoxin reductase (TrxR1), and Relish (Table 1). All expression levels were standardized to two reference genes (β-tubulin and glycerol 3-phosphate dehydrogenase (GPDH)) [43]. Reactions were carried out in 20 μL final volume with 2.5 ng/μL of cDNA, 1x PCR buffer, 0.2 μM of each primer (Table 1), 0.2 mM dNTP, 1.5–5 mM MgCl_2,_ 0,1x SYBR® Green, and 0.02 U platinum Taq DNA polymerase (Invitrogen®) using 40 therm cycles of 15 s at 94 °C, 15 s at 60 °C, and 15 s at 72 °C [72]. SYBR fluorescence was analyzed by Software StepOne 2.0 version (Applied Biosystems). The reactions were performed in duplicates of four to six independent experiments. Dissociation curves at 55 to 99 °C were obtained to confirm the amplification of a single specific product per reaction. The 2^−∆∆CT^ method [73] was used to establish the values of the genic expression.

**Table 1:** Sequence of RT-qPCR primers.

Gene	Left	Right	Flybase gene ID
Nrf2 (cap-n-collar)	AGCGCATCTCGAACAAGTTT	CGTGTTGTTACCCTCGGACT	FBgn0262975
Keap1	CCAACTTCCTCAAGGAGCAG	CGGCGACAAATATCATCCTT	FBgn0038475
NF-κB activating protein-like	CCGCAGAAACCAGAGAGTTC	TGTGCTTTCTCTTGCCCTTT	FBgn0039488
Jafrac1	TGGATCAACACGCCAAGGAA	GGATGCCAGTCTCCTCATCG	FBgn0040309
TrxR1	CGTTCTATTGTGCTGCGTGG	AGCTTGCCATCATCCTGCTT	FBgn0020653
Relish	TTTAGGTGCGGCTCTGCTTT	CTCTCCAGTTTGTGCCGACT	FBgn0014018
GPDH	ATGGAGATGATTCGCTTCGT	GCTCCTCAATGGTTTTTCCA	FBgn0001128
β-Tubulin	ATCCCCAACAACGTGAAGAC	ACCAATGCAAGAAAGCCTTG	FBgn0284243

### Statistical analyses

All results were expressed as mean ± SEM. Data on survival percentage were plotted in the Kaplan-Meier curve and analyzed by Logrank test for trend. Data derived from the biochemical analyses (DCFDA oxidation, GST and AChE activity) were analyzed by Four-way ANOVA [2 with/without VCH × 2 with/without MeHg^+^ × 2 flies ages (four and  six-days-old) x time of kinetic reading (reading (or reaction) time was treated as repeated measures)]. The main effect and lower-order interactions will be discussed only when higher-order interactions were statistically not significant. Data from mRNA levels were analyzed by paired t-test. The results were considered significant when *p* ≤ 0.05.

## Results

### Concentration curve for MeHg^+^

As shown in the Kaplan-Meier survival curve (Fig. 1a), a Logrank test for trend indicated a significant difference among the groups [Chi square (1) = 9.72, *p* = 0.0018]. Exposure of flies to 0.4 mM MeHg^+^ for four days caused a significant reduction in survival when compared to the control group. In contrast, exposure to 0.1 or 0.2 mM MeHg^+^ did not alter the survival rate.

### Percent survival of flies after co-exposure to VCH and MeHg^+^

Flies from both genders were exposed to 0.1% ethanol (control group), 1 mM VCH, 0.2 mM MeHg^+^, and 1 mM VCH + 0.2 mM MeHg^+^ for one or three days. Logrank test for trend indicated no alterations in flies exposed and co-exposed to VCH and MeHg^+^ for one day [Chi square (1) = 1.647, *p* = 0.19] (Fig. 1b). In contrast, three-days co-exposure to VCH and MeHg^+^ led to a significant decrease in percent survival ([Chi square (1) = 16.61, *p* < 0.0001]; Fig. 1c).

### Negative geotaxis

**Fig. 2:**
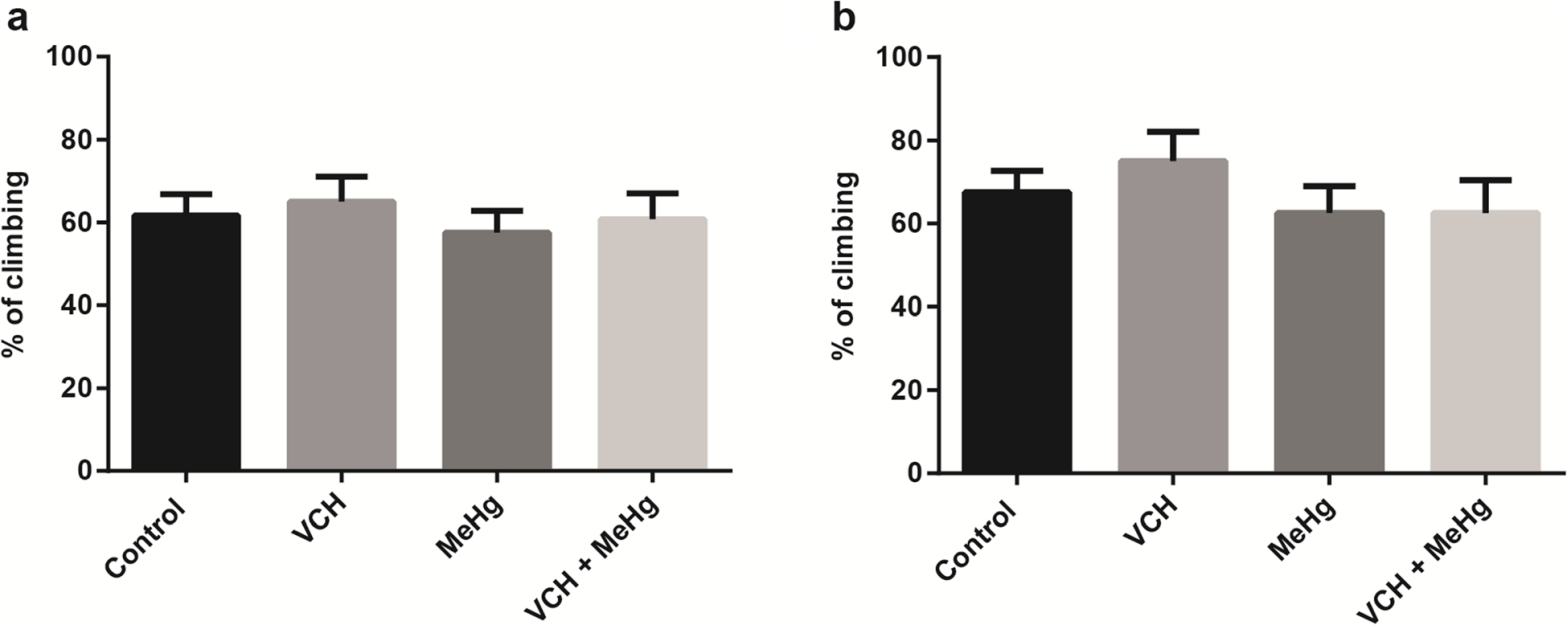
Percent of climbing of flies after exposure and co-exposure to VCH and MeHg^+^ for one (**a**) or three days (**b**). Data were expressed as the mean ± standard error. Results were analyzed by Two-way ANOVA (VCH x MeHg^+^ as independent factors) (*p* > 0.05).

The flies locomotion was not affected by exposure and co-exposure to VCH and MeHg^+^ for one or three days as verified by percent of climbing (Fig. 2a and b, respectively).

### Dichlorofluorescein diacetate oxidation

**Fig. 3:**
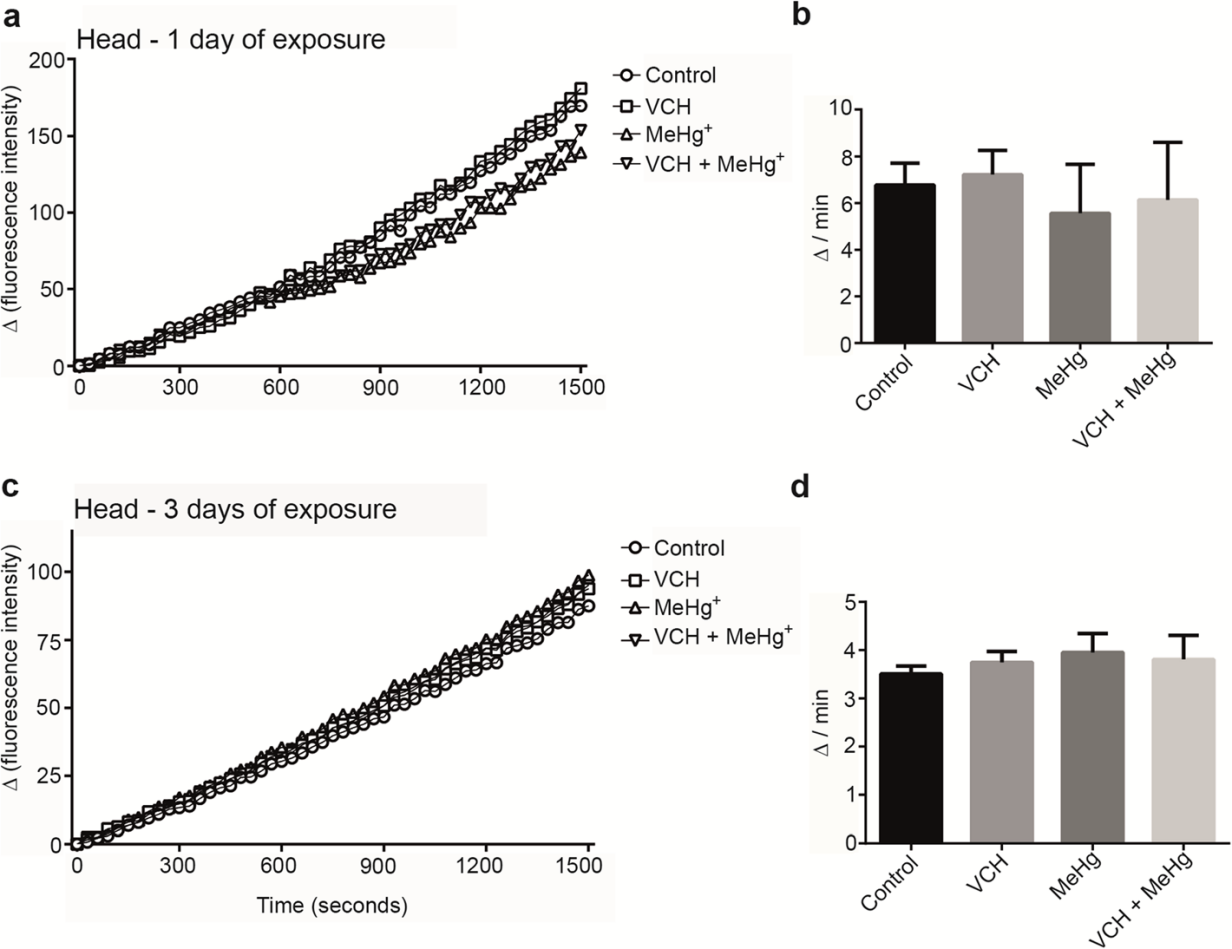
DCFDA oxidation of flies head after exposure and co-exposure to VCH and MeHg^+^ for one (**a** and **b**) or three days (**c** and **d**). Kinetic readings were expressed as the mean in **a** and **c** (standard errors were omitted for better viewing) and delta per minute as mean ± standard error in **b** and **d**). Results were analyzed by four-way ANOVA (VCH x MeHg^+^ x age x time of kinetic reading which was treated as repeated measures) and were considered significantly different when *p* < 0.05

For DCFDA oxidation, the between-subjects part of the four-way ANOVA revealed a significant interaction between MeHg^+^ x age in the flies’ head. The interaction was significant, because, after one day of exposure, MeHg^+^ significantly decreased the oxidation of DCFDA in the head, whereas DFCDA oxidation increased after three days of exposure [F(1,88) = 4.96, *p* < 0.03 (Fig. 3 a-d and Table 2)]. The within-subjects part of the ANOVA indicated a significant MeHg^+^ x age x time of kinetic reading. The analysis of DCFDA oxidation as a function of reaction time (Fig. 3a and c) confirmed that the increase in oxidation of DCFDA decreased after one day of exposure to MeHg^+^, but increased after three days of exposure relative to the control group [F(49,4312) = 5.41, *p* < 0.001 (Table 2)]. The main and interactive effects associated with VCH exposure were not significant (data were not shown).

**Table 2:** Statistical analyses of DCFDA oxidation

Tissue	ANOVA	Interaction	DF	F	P
Head	Between-subjects	MeHg^+^ x age^a^	1,88	4.96	0.03
	Within-subjects	MeHg^+^ x age x time^b^	49,4312	5.41	< 0.001
Body	Between-subjects	VCH x MeHg^+^ x age	1,88	4.97	0.03
	Within-subjects	Time x VCH x MeHg^+^ x age	49,4312	3.74	< 0.001

^a^At the end of exposure periods (one or three days) flies had ages of four and six-days-old, respectively. ^b^ Reaction time

**Fig. 4:**
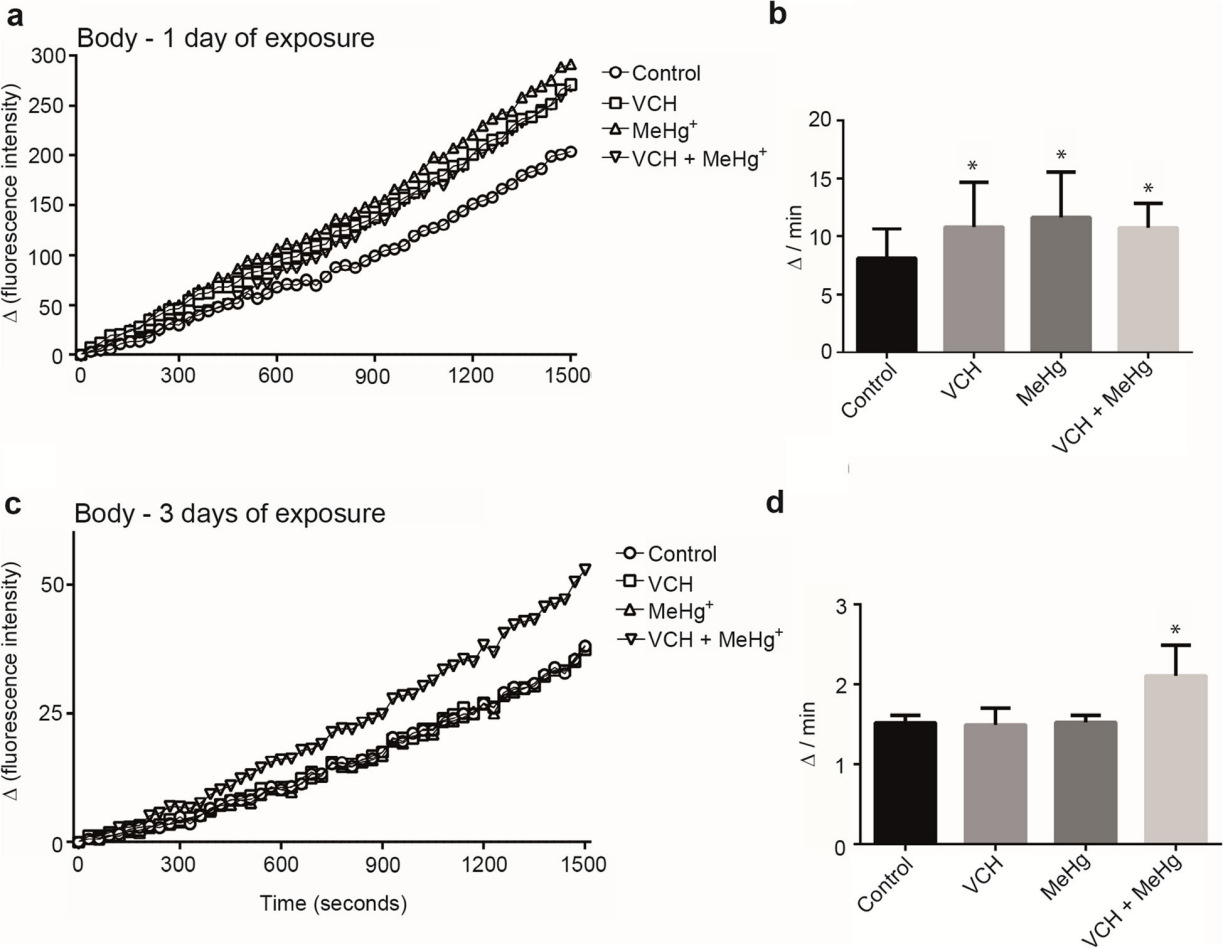
DCFDA oxidation of flies body after exposure and co-exposure to VCH and MeHg^+^ for one (**a** and **b**) or three days (**c** and **d**). Kinetic readings were expressed as the mean in **a** and **c** (standard errors were omitted for better viewing) and delta per minute as mean ± standard error in **b** and **d**. Results were analyzed by four-way ANOVA (VCH x MeHg^+^ x age x time of kinetic reading which was treated as repeated measures) and were considered significantly different when *p* < 0.05

Multifactorial ANOVA revealed a significant third-order interaction (VCH x MeHg^+^ x age) in the body. This interaction was significant, because after one day of exposure to VCH, MeHg^+^ or VCH + MeHg^+^ the oxidation of DCFDA was increased, whereas after three days of exposure only simultaneous exposure to VCH and MeHg^+^ increased the oxidation of DCFDA [F(1,88) = 4.97; *p* < 0.03 (Fig. 4a-d and Table 2)]. The within-subjects part of the ANOVA indicated a significant fourth-order interaction (reaction time x VCH x MeHg^+^ x age). The analysis of DCFDA oxidation as a function of reaction time (Fig. 4a and c) demonstrated that the increase in oxidation of DCFDA was inherent to all treatments after one day of exposure, but it was increased only upon the simultaneous exposure to VCH and MeHg^+^ after three days of exposure [F(49,4312) = 3.74; *p* < 0.001 (Table 2)].

### Glutathione S-transferase activity

**Fig. 5:**
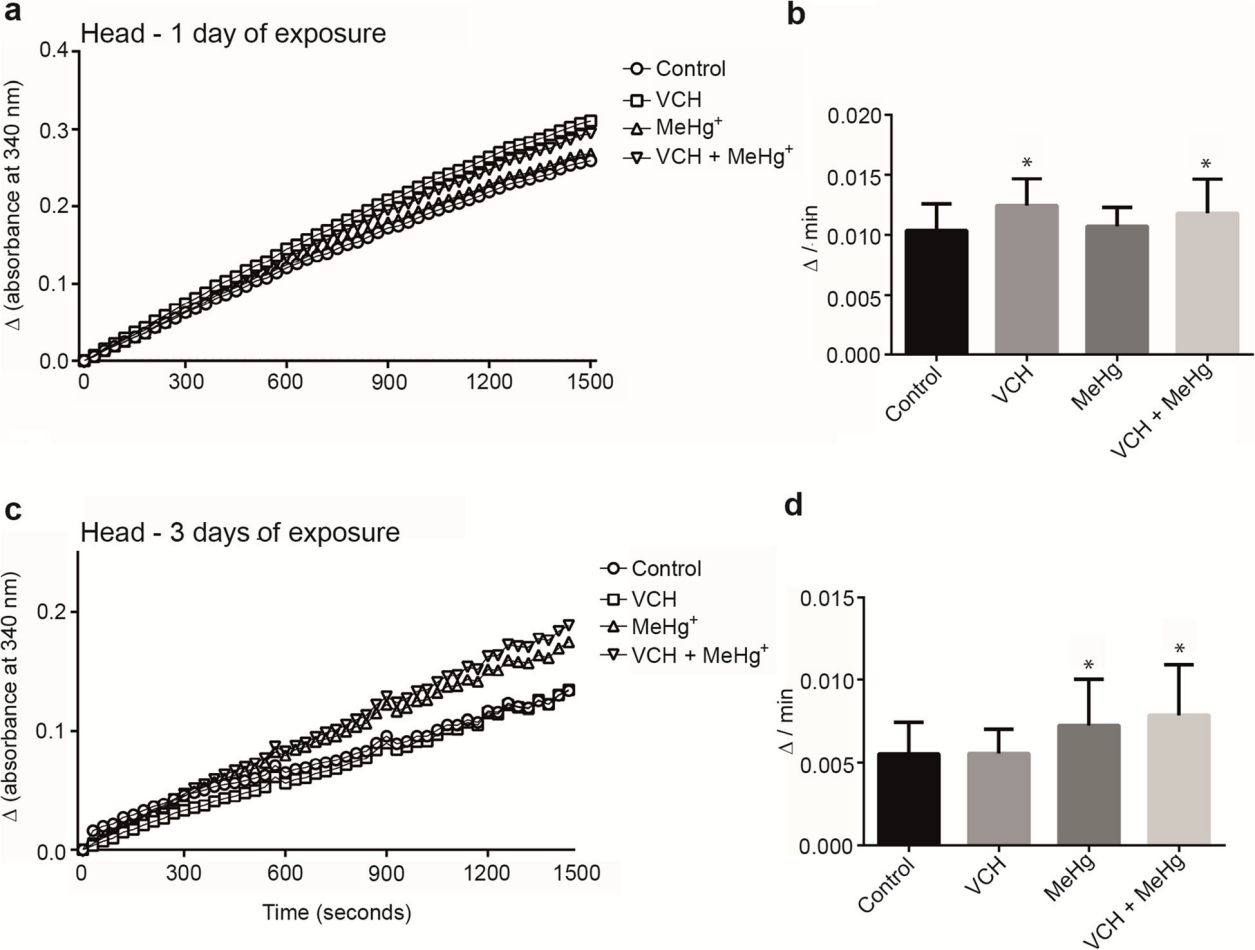
GST activity of flies head after exposure and co-exposure to VCH and MeHg^+^ for one (**a** and **b**)or three days (**c** and **d**). Kinetic readings were expressed as the mean in **a** and **c** (standard errors were omitted for better viewing) and delta per minute as mean ± standard error in **b** and **d**. Results were analyzed by four-way ANOVA (VCH x MeHg^+^ x age x time of kinetic reading which was treated as repeated measures) and were considered significantly different when *p* < 0.05

For GST activity in the flies’ head, the between-subjects part of the four-way ANOVA revealed a significant main effect of age, given that the younger flies (one day of exposure) exhibited higher head GST activity than older flies (three days of exposure) [F(1,88) = 94.78; *p* < 0.001 (Fig. 5a-d and Table 3)]. The within-subjects part of the ANOVA indicated a significant reaction time x VCH interaction. The analysis of head GST activity as a function of reaction time (Figs. 5a and c) confirmed that the VCH increased GST activity after one day of exposure, but had no effect after three days of exposure [F(47,4136) = 5.55; *p* < 0.001 (Table 3)]. There was also a third-order interaction (reaction time x MeHg^+^ x age).

**Table 3:** Statistical analyses of GST activity

Tissue	ANOVA	Interaction	DF	F	p
Head	Between-subjects	Age^a^	1,88	94.78	< 0.001
	Within-subjects	Time^b^x VCH	47,4136	5.55	< 0.001
		Time x MeHg^+^ x age	47,4136	6.62	< 0.001
Body	Between-subjects	Age	1,88	300.19	< 0.001
		VCH x MeHg^+^	1,88	4.62	0.03
	Within-subjects	Time x VCH x MeHg^+^ x age	47,4136	1.96	< 0,001

^a^At the end of exposure periods (one or three days) flies had ages of four and six-days-old, respectively. ^b^ Reaction time

**Fig. 6:**
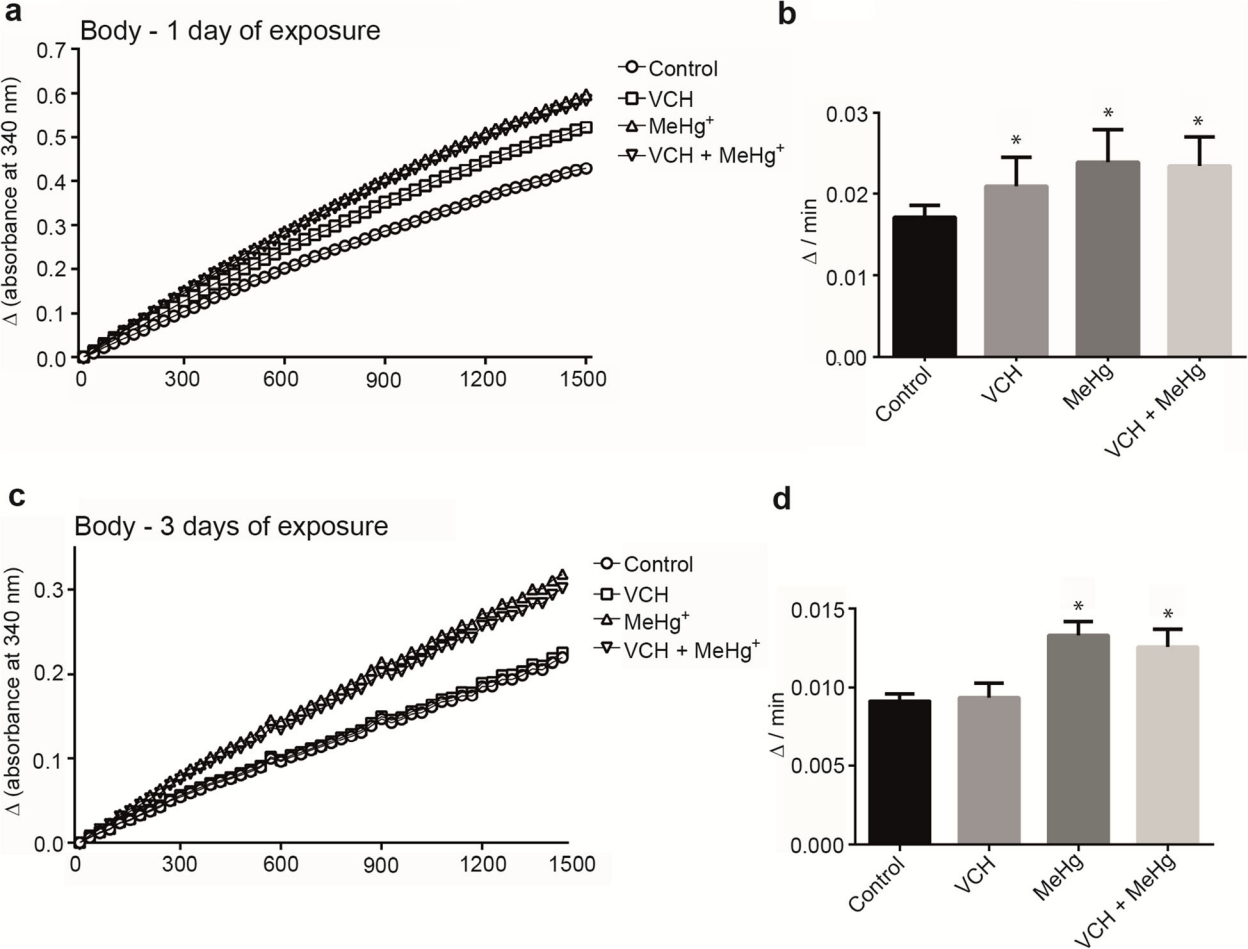
GST activity of flies body after exposure and co-exposure to VCH and MeHg^+^ for one (**a** and **b**)or three days (**c** and **d**). Kinetic readings were expressed as the mean in **a** and **c** (standard errors were omitted for better viewing) and delta per minute as mean ± standard error in **b** and **d**. Results were analyzed by four-way ANOVA (VCH x MeHg^+^ x age x time of kinetic reading which was treated as repeated measures) and were considered significantly different when *p* < 0.05

In the body, the between-subjects part of the four-way ANOVA revealed a significant main effect of age, because the GST activity of younger flies (one day of exposure) was higher than in older flies (three days of exposure) [F(1,88) = 300.19; *p* < 0.001 (Fig. 6a-d and Table 3)]. There was also an interaction between VCH x MeHg^+^ in the body of the flies. The interaction was significant, because after one day of treatment with VCH, MeHg^+^, and VCH + MeHg^+^ GST increased, whereas only MeHg^+^ increased it after three days of exposure [F(1,88) = 4.62; *p* > 0.04 (Table 3)]. The within-subjects part of the ANOVA showed a significant fourth-order interaction (reaction time x VCH x MeHg^+^ x age). The analyses of GST activity as a function of reaction time (Fig. 6a and c) demonstrated increased GST activity in all treatments after one day of exposure, but it was increased only upon three days of exposure to MeHg^+^ [F(47, 4136) = 1.96; p < 0.001 (Table 3)].

### Acetylcholinesterase activity

**Fig. 7:**
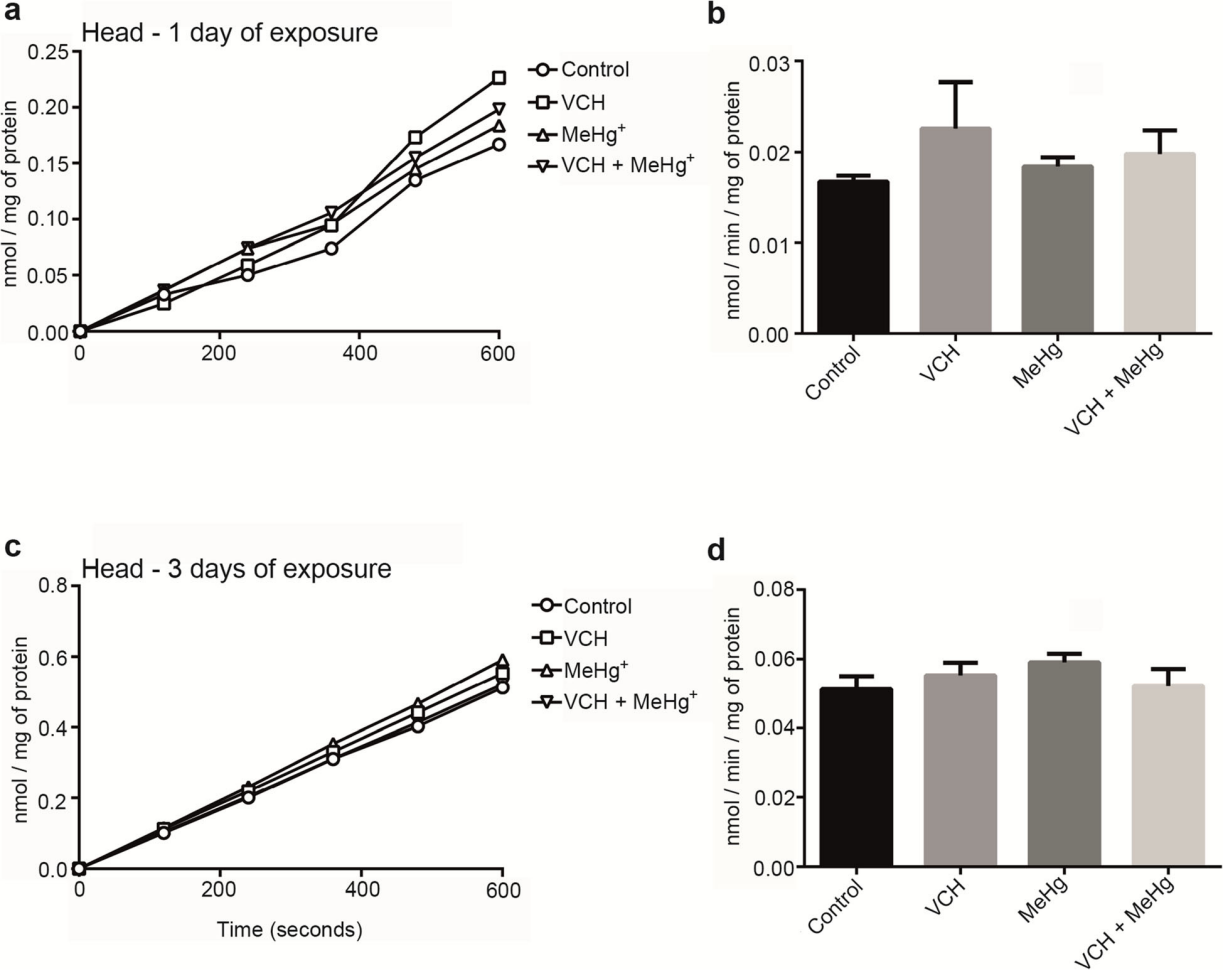
AChE activity of flies head after exposure and co-exposure to VCH and MeHg^+^ for one (**a** and **b**)or three days (**c** and **d**). Kinetic readings were expressed as the mean in **a** and **c** (standard errors were omitted for better viewing) and delta per minute as mean ± standard error in **b** and **d**. Results were analyzed by four-way ANOVA (VCH x MeHg^+^ x age x time of kinetic reading which was treated as repeated measures) and were considered significantly different when *p* < 0.05

The between-subjects part of the four-way ANOVA on AChE activity in the head of flies revealed a significant main effect of age, as the AChE activity of younger flies (one day of exposure) was lower than in older flies (three days of exposure) [F(1,88) = 216.02; p < 0.001 (Fig. 7a-d and Table 4)]. The analysis of AChE activity as a function of reaction time (Fig. 7a and c) indicated a third-order interaction (reaction time x VCH x MeHg^+^), since VCH increased the AChE activity after one day of exposure, but returned to basal levels after three days of exposure. In contrast, MeHg^+^ caused an increase in AChE activity after three days of exposure, but not after one day of exposure (where VCH tended to increase the enzyme activity) [F(4,352) = 2.6; *p* < 0.04 (Table 4)].

**Table 4:** Statistical analyses of AChE activity

Tissue	ANOVA	Interaction	DF	F	p
Head	Between-subjects	Age^a^	1,88	216,02	< 0.001
	Within-subjects	Time^b^ x VCH x MeHg^+^	4352	2,60	0.04

^a^At the end of exposure periods (one or three days) flies had ages of four and six-days-old, respectively. ^b^ Reaction time

### Gene expression

**Fig. 8:**
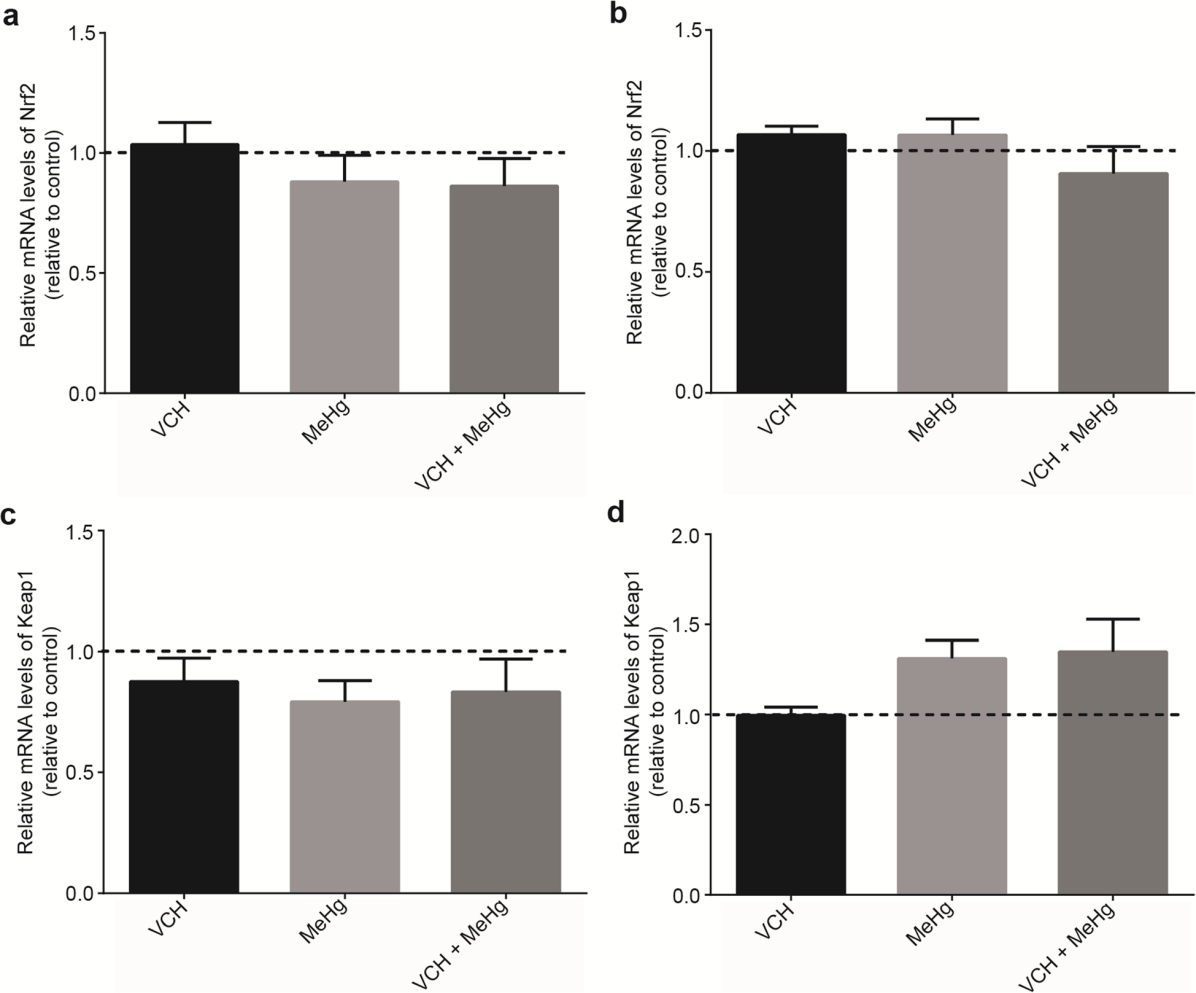
Expression of the genes encoding Nrf2 and Keap1. mRNA levels of Nrf2 in the head (**a**) and body (**b**), as well as, Keap 1 in the head (**c**) and body (**d**) of *D. melanogaster* after exposure and coexposure to VCH and MeHg^+^ for three days. Data are expressed as the mean ± standard error. Results were analyzed by paired t-test and were considered significantly different when *p* < 0.05.

**Fig. 9:**
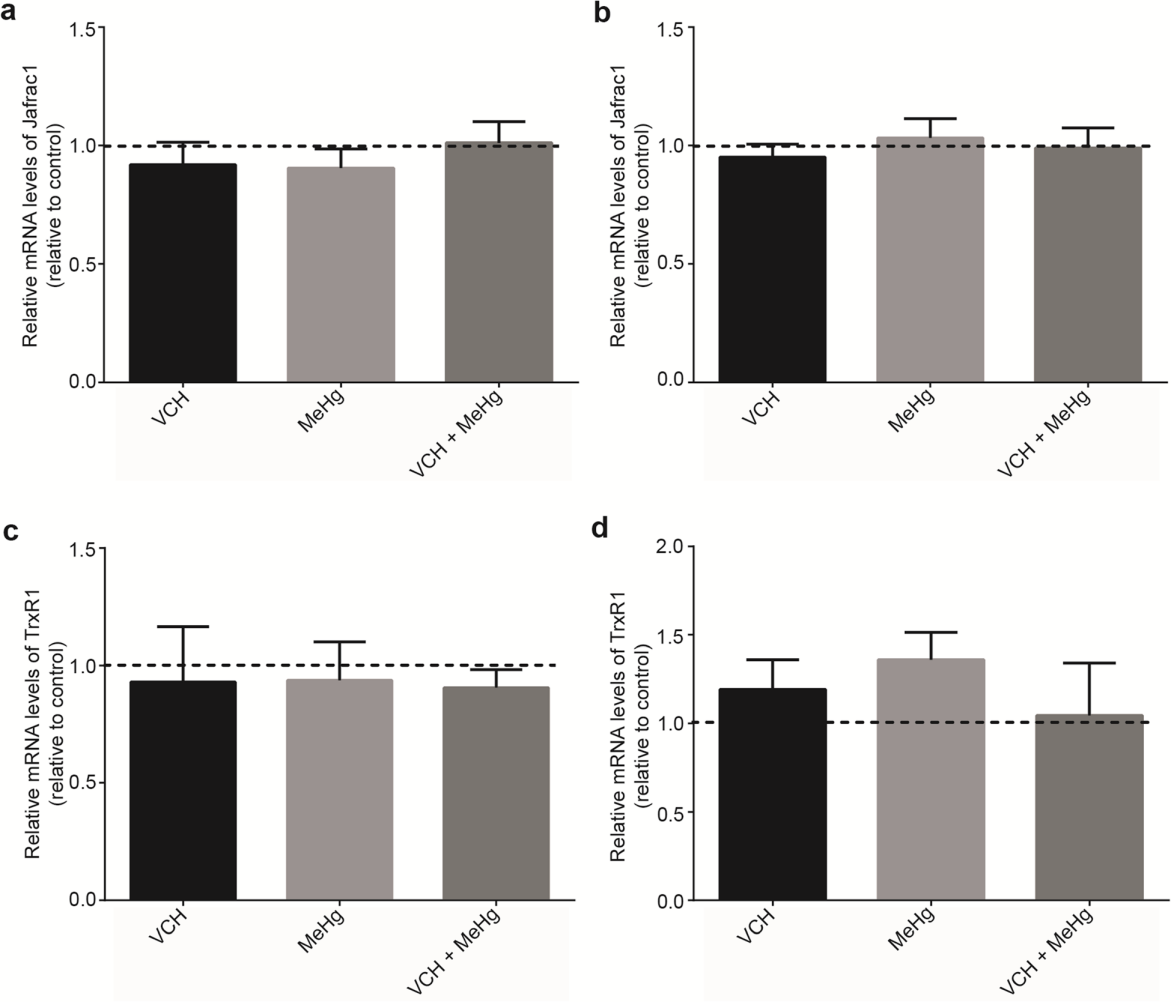
Expression of the genes encoding Jafrac1 and TrxR1. mRNA levels of Jafrac1 in the head (**a**) and body (**b**), TrxR1 in the head (**c**) and body (**d**) of *D. melanogaster* after exposure and co-exposure to VCH and MeHg^+^ for three days. Data are expressed as the mean ± standard error. Results were analyzed by paired t-test and were considered significantly different when *p* < 0.05

**Fig. 10:**
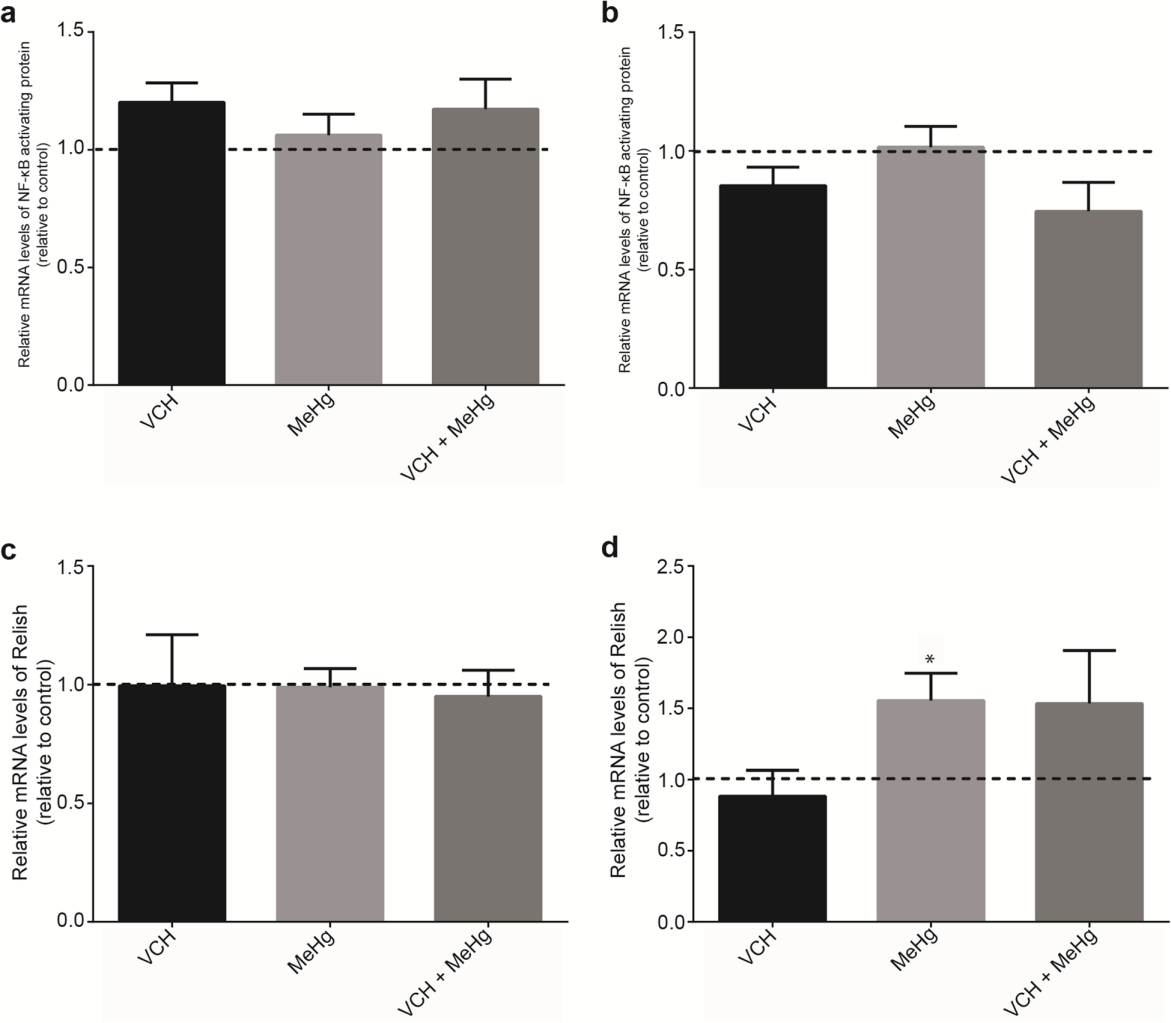
Expression of the genes encoding NF-κB activating protein and Relish. mRNA levels of NF-κB activating protein in the head (**a**) and body (**b**), Relish in the head (**c**) and body (**d**) of *D. melanogaster* after exposure and co-exposure to VCHand MeHg^+^ for three days. Data are expressed as the mean ± standard error. Results were analyzed by paired t-test and were considered significantly different when *p* < 0.05

Expression of genes involved in oxidative stress and inflammatory responses of flies upon exposure and coexpure to VCH and MeHg^+^ for three days are shown in Figs. 8, 9 and 10. Figure 8 depicts the expression of Nrf2 and Keap1 in the head (Fig. 8a and c) and the body (Fig. 8b and d). Treatment with VCH, MeHg^+^ or VCH + MeHg^+^ did not alter mRNA levels of Nrf2 and Keap1 in both head and body.

mRNA levels of genes related to oxidative stress (Jafrac1 and TrxR1) is shown in Fig. 9. Exposure to VCH, MeHg^+^ or VCH + MeHg^+^ did not alter Jafrac1 (Fig. 9a and b) and TrxR1 (Fig. 9c and d) expression levels in both tissues.

Expression of the NF-κB activating protein-like (Fig. 10a and b) and Relish (Fig. 10c and d) genes were evaluated. MeHg^+^ caused a significant upregulation of Relish in body of flies (*p* = 0.03). The treatments unaltered the mRNA levels of NF-κB activating protein-like (head and body) and Relish (head).

## Discussion

To maintain redox homeostasis in aerobic organisms, a complex interplay between various classes of proteins, such as transcription factors, antioxidant enzymes and electron donating molecules must occur [74]. Reactive oxygen or nitrogen species are continuously formed as byproducts of physiologic reactions [75]. However, xenobiotics can trigger an imbalance in redox homeostasis, causing either direct damage to biomolecules or directly promoting oxidative stress. Exposure to electrophilic xenobiotics can disrupt the cellular redox balance and change the expression of various classes of genes [76, 77].

Here we observed that VCH, MeHg^+^, or VCH + MeHg^+^ caused oxidative stress in the body of flies after one day of exposure. Also, co-exposure to VCH and MeHg^+^ for three days caused oxidative stress in the flies’ body. These results corroborate earlier studies where individual exposures to MeHg^+^ or VCH were shown to increase reactive species production in various animal models [16, 43, 44, 78–80].

Since reactive metabolites of VCH and MeHg^+^ can form adducts with proteins containing soft nucleophile centers, we determined the activity of GSTs. The latter are considered biomarkers of toxicity to environmental contaminants [81–83]. GSTs are a family of enzymes that catalyze phase II detoxification reactions and conjugate reduced GSH with electrophilic molecules [84–88]. In the head of flies, VCH led to increased GST activity after one day of exposure, while MeHg^+^ and VCH + MeHg^+^ increased GST activity only after three days of exposure. In the body, VCH, MeHg^+^, and VCH + MeHg^+^ increased the enzyme’s activity after one day of exposure, and MeHg^+^ and VCH + MeHg^+^ increased it after three days of exposure. GSTs catalyze the conjugation of VCH metabolites [39–41] with GSH, preventing their interaction with target proteins. The increase in GST activity may be related to an adaptive response that counteracts the electrophile toxicity inherent to VCH. An analogous process of detoxification might occur with GSH and MeHg^+^ [33, 38]. However, there are contradictory studies, where exposure to MeHg^+^ induces either an increase or decrease in GST activity, dependent upon the experimental model and the tissues analyzed [89–91]. Here, we observed an intricate pattern of changes in total GST activity, which was dependent upon the tissue, the type of chemical, and the period of exposure. Notably, the enzyme activity increased after exposure to VCH, MeHg^+^, and VCH + MeHg^+^. This likely reflects a compensatory response to counteract the toxicity of both xenobiotics, consistent with recent observations in *D. melanogaster*, where several GST isoforms (GSTD1, GSTE1, and GSTS1) have been shown to protect larvae during pupal development and knockdown of GSTE1, and GSTS1 isoforms increased the susceptibility to MeHg^+^ toxicity [38].

Previously, we and other researches have shown that AChE can be used as a biomarker of exposure to xenobiotics [92–95]. Early studies have demonstrated the inhibition of AChE after exposure to MeHg^+^ in flies, cockroaches, and rats [96–98], as well as after exposure to VCH in flies [43], yet, here we failed to observe inhibition of the enzyme. Inhibition of AChE activity can be associated with impairments in fly locomotion [43, 44]. The flies’ behavior was unaltered, which is consistent with unaltered AChE activity. Probably the exposure period and/or doses of the toxicants were not sufficient to alter this enzyme and, consequently, to reflect on the behavior of the flies.

Among proteins that are related to oxidative stress, KEAP1 represses the translocation of the NRF2 to the nucleus [99, 100]. Once in the nucleus, NRF2 binds to the antioxidant response element (ARE or electrophile response element or EpRE), an enhancer cis-element, stimulating the expression of stress-responsive genes [101]. In *D. melanogaster,* this mechanism is analogous to the one reported in mammals. NRF2 in flies is referred to as CncC, and it is part of the transcription factor family Cap’n’collar [102, 103], which activates many cytochrome P450 and GST coding genes [103–105]. The gene CG3962 encodes a Kelch protein, which is similar to KEAP1 [103]. Nrf2 and Keap1 expression in the flies were not altered upon three-days exposure to VCH and MeHg^+^. Since MeHg^+^ or VCH increased GST activity, we expected to find an increase in the expression of Nrf2 and Keap1. Indeed, literature data have indicated an essential role for this transcription factor in the toxicity of MeHg^+^ [106–108]. However, their expression did not differ from control flies. It is plausible that the expression of several isoforms of GST that are not under the control of NRF2 or that the activation of this transcription factor has occurred in a KEAP1-independent form. Next, we tested genes directly related to oxidative stress metabolism. Peroxiredoxins are a family of enzymes that reduce H_2_O_2_ or organic peroxides to H_2_O or R-OH, respectively [54]. *D. melanogaster* expresses seven peroxiredoxins, including Jafrac1 that is homolog to Prx2 of mammals [109]. Herein, the Jafrac1 expression was not altered by three-days exposure to VCH and MeHg^+^. Thioredoxins (Trx) are a family of small proteins with redox active thiol groups, which can donate reducing equivalents to various proteins. The oxidation of reduced Trx [Trx(−SH)_2_] forms the oxidized Trx [Trx(S)_2_] that may undergo further reduction by NADPH-dependent TrxR, thus restoring Trx [Trx(−SH)_2_] [110]. Here, the mRNA levels of TrxR1 were not altered by VCH and MeHg^+^. Finally, to understand whether simultaneous exposure to VCH and MeHg^+^ might activate select inflammatory responses, we analyzed the mRNA levels of NF-κB activating protein and Relish. NF-κB belongs to a ubiquitous dimer family that regulates the expression of a large number of genes involved in immune regulation, inflammatory responses, and anti-apoptotic effects [111]. In the present study, after three-days exposure, MeHg^+^ induced upregulation of the Relish in the body of the flies. In *D. melanogaster* three NF-κB/Rel proteins have been identified, e.g., Relish, Dif, and Dorsal [112, 113]. Exogenous environmental factors selectively activate the NF- κB/Rel proteins of *D. melanogaster*. Here we observed the upregulation of Relish in the body of flies exposed to MeHg^+^ for three days. Since Relish is involved in inflammatory processes in flies, we speculate that MeHg^+^ may activate inflammatory responses via this route.

One limitation of our study is the absence of data about the VCH and MeHg^+^ absorption in flies. Concerning the distribution of MeHg^+^, large amounts of MeHg^+^ were found in adult fly’s brain after oral exposure [114]. As for VCH, there are no data in the literature on the rate of absorption and distribution in living organisms. Consequently, future studies have to be done to clarify these topics about the distribution of VCH and MeHg^+^ in flies.

## Conclusions

The results presented herein indicate that VCH and MeHg^+^, when co-administered, did not potentiate their individual effects in flies. In general, MeHg^+^ modified a higher number of endpoints than did VCH, likely reflecting its higher electrophilicity. Our findings established that Relish might be an early molecular marker of MeHg^+^ toxicity. Furthermore, the response of GSTs to both toxicants indicates that the study of the expression of specific isoforms of this gene family might contribute to better understanding the molecular mechanisms involved in the toxicity of two important environmental contaminants. Additional genes associated with oxidative stress and inflammatory response should be studied as possible toxicity markers. The development and standardization of biomarkers of exposure for the detection of early toxicological changes induced by low concentration of toxic agents are criticalin predicting and preventing chronic exposure effects.

## Data Availability

The datasets analyzed during the current study are available from the corresponding author on reasonable request.

## Abbreviations

AChE: Acetylcholinesterase
DCFDA: 2,7-dichlorofluorescein diacetate
GSH: Glutathione
GST: Glutathione S-transferase
Keap1: Kelch-like erythroid cell-derived associated protein 1
MeHg^+^: Methylmercury
Nrf2: Nuclear factor-erythroid 2-related factor 2
TrxR: Thioredoxin reductase
VCD: 4-vinylcyclohexene diepoxide
VCH: 4-vinylcyclohexene
